# Multi-parametric evaluation of the white matter maturation

**DOI:** 10.1007/s00429-014-0881-y

**Published:** 2014-09-03

**Authors:** S. Kulikova, L. Hertz-Pannier, G. Dehaene-Lambertz, A. Buzmakov, C. Poupon, J. Dubois

**Affiliations:** 1 UMR 1129 NeuroSpin/UNIACT, INSERM-CEA, Gif-sur-Yvette, France; 2 UMR 992 NeuroSpin/UNICOG INSERM-CEA, Gif-sur-Yvette, France; 3 LORIA, CNRS-Inria Nancy Grand Est-Université de Lorraine, Nancy, France; 4 NeuroSpin/UNIRS CEA-Saclay, Gif-sur-Yvette, France; 5 CEA/SAC/DSV/I2BM/NeuroSpin, Bât 145, point courrier 156, 91191 Gif-sur-Yvette, France

**Keywords:** Mahalanobis distance, White matter, Brain development, Bundles, Infants, T1 and T2 relaxometry, Diffusion tensor Imaging DTI

## Abstract

In vivo evaluation of the brain white matter maturation is still a challenging task with no existing gold standards. In this article we propose an original approach to evaluate the early maturation of the white matter bundles, which is based on comparison of infant and adult groups using the Mahalanobis distance computed from four complementary MRI parameters: quantitative qT1 and qT2 relaxation times, longitudinal λ_║_ and transverse λ_⊥_ diffusivities from diffusion tensor imaging. Such multi-parametric approach is expected to better describe maturational asynchrony than conventional univariate approaches because it takes into account complementary dependencies of the parameters on different maturational processes, notably the decrease in water content and the myelination. Our approach was tested on 17 healthy infants (aged 3- to 21-week old) for 18 different bundles. It finely confirmed maturational asynchrony across the bundles: the spino-thalamic tract, the optic radiations, the cortico-spinal tract and the fornix have the most advanced maturation, while the superior longitudinal and arcuate fasciculi, the anterior limb of the internal capsule and the external capsule have the most delayed maturation. Furthermore, this approach was more reliable than univariate approaches as it revealed more maturational relationships between the bundles and did not violate a priori assumptions on the temporal order of the bundle maturation. Mahalanobis distances decreased exponentially with age in all bundles, with the only difference between them explained by different onsets of maturation. Estimation of these relative delays confirmed that the most dramatic changes occur during the first post-natal year.

**Electronic supplementary material:**

The online version of this article (doi:10.1007/s00429-014-0881-y) contains supplementary material, which is available to authorized users.

## Introduction

Maturation of the brain white matter is a complex process, which lasts from the third trimester of pregnancy until late adolescence, and proceeds in an asynchronous manner across cerebral regions (Yakovlev and Lecours 1967). Early *post-mortem* studies have shown that different white matter regions myelinate over different periods of time and at different rates, from the central regions to the periphery (Flechsig 1920). For instance, certain projection bundles (e.g. cortico-spinal and spino-thalamic tracts) mature before association bundles related to cognitive functions such as language (e.g. arcuate fasciculus) (Brody et al. 1987; Kinney et al. 1988). However, *post-mortem* studies have insurmountable limitations: they do not allow making correlations between anatomical and functional changes during maturation and provide “region-specific” rather than “bundle-specific” information. In vivo imaging is thus indispensable for understanding both normal and pathological brain development, but it remains a challenging task in unsedated infants.

Conventional Magnetic Resonance Imaging (MRI) studies, using T1- and/or T2-weighted images have confirmed that different white matter regions acquire “myelinated” appearance in a specific temporal order (Paus et al. 2001): first, in pons and cerebral peduncles, then in the optic radiations, the posterior limb of the internal capsule and the splenium of the corpus callosum, followed by the anterior limb of the internal capsule, the genu of the corpus callosum and finally, by the white matter of the occipital, frontal, parietal and temporal lobes. Whereas these studies provided only qualitative description of the white matter maturation, alternatives have been recently proposed with the quantitative mapping of the relaxation times qT1 and qT2 (Deoni et al. 2005) and with diffusion tensor imaging (DTI), which computes distinct parameters (mean <D>, longitudinal λ_║_ and transverse λ_⊥_ diffusivities, fractional anisotropy FA (Le Bihan and Johansen-Berg 2012)) that can be quantified along the white matter bundles reconstructed using fiber tracking technics (Mori and van Zijl 2002).

All these parameters are known to change with age and are thought to reflect different maturational processes (Dubois et al. 2014a). qT1 mostly depends on the brain water and lipid contents (Steen et al. 1997), whereas qT2 mostly depends on water content and iron accumulation (Engelbrecht et al. 1998); both qT1 and qT2 decrease with age but changes in qT2 (associated with “true myelination”) are known to start later than in qT1 (associated with “pre-myelination”) (Barkovich et al. 1988).

Changes in the DTI parameters are more complex: they depend on the bundle maturational stage and are thought to reflect various processes such as organization of the nervous fibers into bundles, membrane proliferation in the intra- and extra-cellular space (‘‘pre-myelination’’) and myelination (Dubois et al. 2008, 2014a). Some fractional anisotropy can be observed even early on in poorly myelinated bundles of the premature newborns because of the tight organization of the fibers into bundles (Hüppi et al. 1998). With the decrease in water content and the increase in membrane density, all diffusivities decrease. During fiber myelination, fractional anisotropy increases due to a decrease in transverse diffusivity contrasting with constant longitudinal diffusivity.

Although it is possible to make inferences on bundles maturation on the basis of only one MRI or DTI parameter, the univariate approaches may not be efficient to discriminate bundles that are at different maturational stages. For example, the approach of Dubois et al. (2008), based on DTI indices, was supported in only 8 out of 11 bundles, facing problems in classification of the corpus callosum, external capsule and uncinate fasciculus. Thus, taking advantage of the complementary dependencies of the MRI parameters on maturational processes and considering multi-parametric maturational models should enable better characterization of the bundles maturation.

To evaluate a maturational stage of a given infant bundle at a certain age, one needs to compare the parameters characterizing that bundle with the typical values for the same bundle in an adult group, i.e. to compute the “maturational distance” between current and adult stages. Since MRI and DTI parameters are also known to vary across different bundles in the adult brain and to have different scales for different parameters (Dubois et al. 2008), their normalization is required before comparison. Furthermore, a well-designed “maturational distance” should take into account the inter-subject variability of the parameters in the adult population as well as their correlations: the difference between adult and infant values may be important or not, depending on whether it is or not within the range of the parameters variability in the adult population.

According to all these constraints, we introduce here a novel strategy to reliably describe and efficiently compare the bundles maturation in infants from 1 to 5 months of age. This strategy is based on estimation of the Mahalanobis distance between the multi-parametric vectors of four parameters (qT1, qT2, λ_║,_ λ_⊥_) describing bundles in infant and adult groups, and it is compared with univariate approaches. In addition to ordering the bundles according to their relative maturation, our approach suggests a general description of the maturation that allows estimating the relative maturational delays between the bundles.

## Materials and methods

### Subjects

This research study was performed on 17 healthy infants born at term (7 girls, 10 boys), with a maturational age (i.e. chronological age corrected for gestational age at birth) between 3 and 21 weeks. Infants were compared to an adult group of 13 healthy subjects (6 women, 7 men, mean age: 22.4 ± 1.6 years). Additionally, a 34-week-old girl (almost 8 months) was imaged for the model evaluation at an older age. None of the subjects displayed any neurodevelopmental problems or any brain abnormalities observed on MR images. The study protocol was approved by the regional ethical committee for biomedical research; all parents and adult subjects gave written informed consents. Infants were spontaneously asleep during MR imaging. Particular precautions were taken to minimize noise exposure, by using customized headphones and covering the magnet tunnel with special noise protection foam.

### MRI acquisitions

Data acquisition was performed on a 3T MRI system (Tim Trio, Siemens Medical Systems, Erlangen, Germany), equipped with a whole body gradient (40 mT/m, 200 T/m/s) and a 32-channel head coil. Interleaved axial slices covering the whole brain were imaged with a 1.8-mm isotropic spatial resolution (FOV = 23 × 23 cm^2^, matrix = 128 × 128) using EPI single-shot spin-echo (SE) sequences (50 slices for infants; 70 for adults). For DTI, a DW-SE-EPI sequence was used with 30 orientations of diffusion gradients with *b* = 700 s mm^−2^ (+*b* = 0 volume): TE = 72 ms, TR = 10 s (TR = 14 s for adults), parallel imaging GRAPPA factor 2, partial Fourier sampling factor 6/8, leading to an acquisition time of 5 min 40 s (7 min 56 s for adults). For qT1 mapping, an inversion recovery (IR) SE-EPI sequence was used with eight different values of inversion time (TI = 250→1,500 ms each step 250 ms + TI = 2,000, 2,500 ms): TE = 38 ms, TR = TI + 15 s (TR = TI + 21 s for adults), partial Fourier sampling factor 5/8, leading to an acquisition time of 2 min 11 s (3 min 03 s for adults). For qT2 mapping, an SE-EPI sequence was used with 8 different values of echo time (TE = 50→260 ms each step 30 ms): TR = 15.5 s (TR = 21.7 s for adults), parallel imaging GRAPPA factor 2, partial Fourier sampling factor 6/8, leading to an acquisition time of 2 min 51 s (4 min for adults).

### Data post-processing

**Fig. 1:**
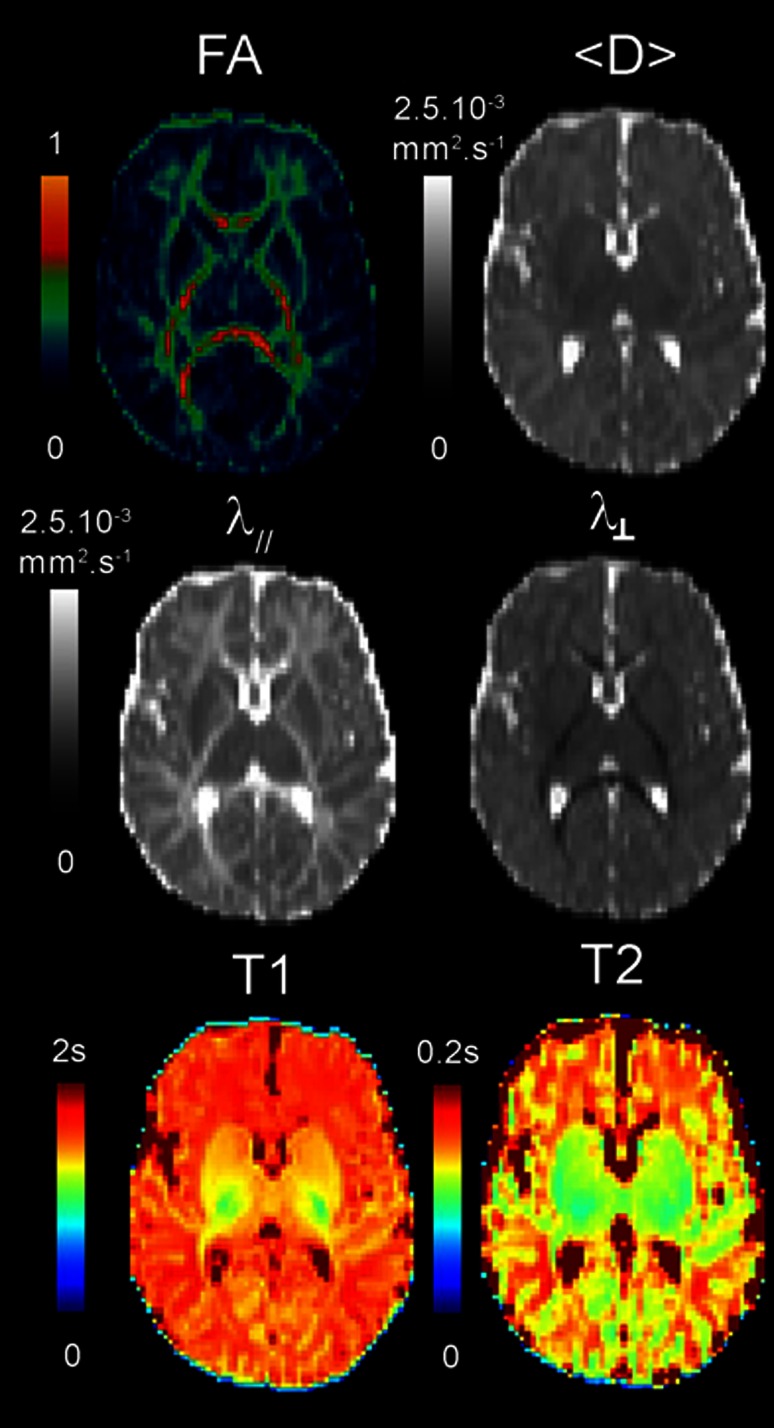
Quantitative maps of MRI parameters. Maps of DTI parameters and relaxation times are presented for a 6-week-old infant

**Fig. 2:**
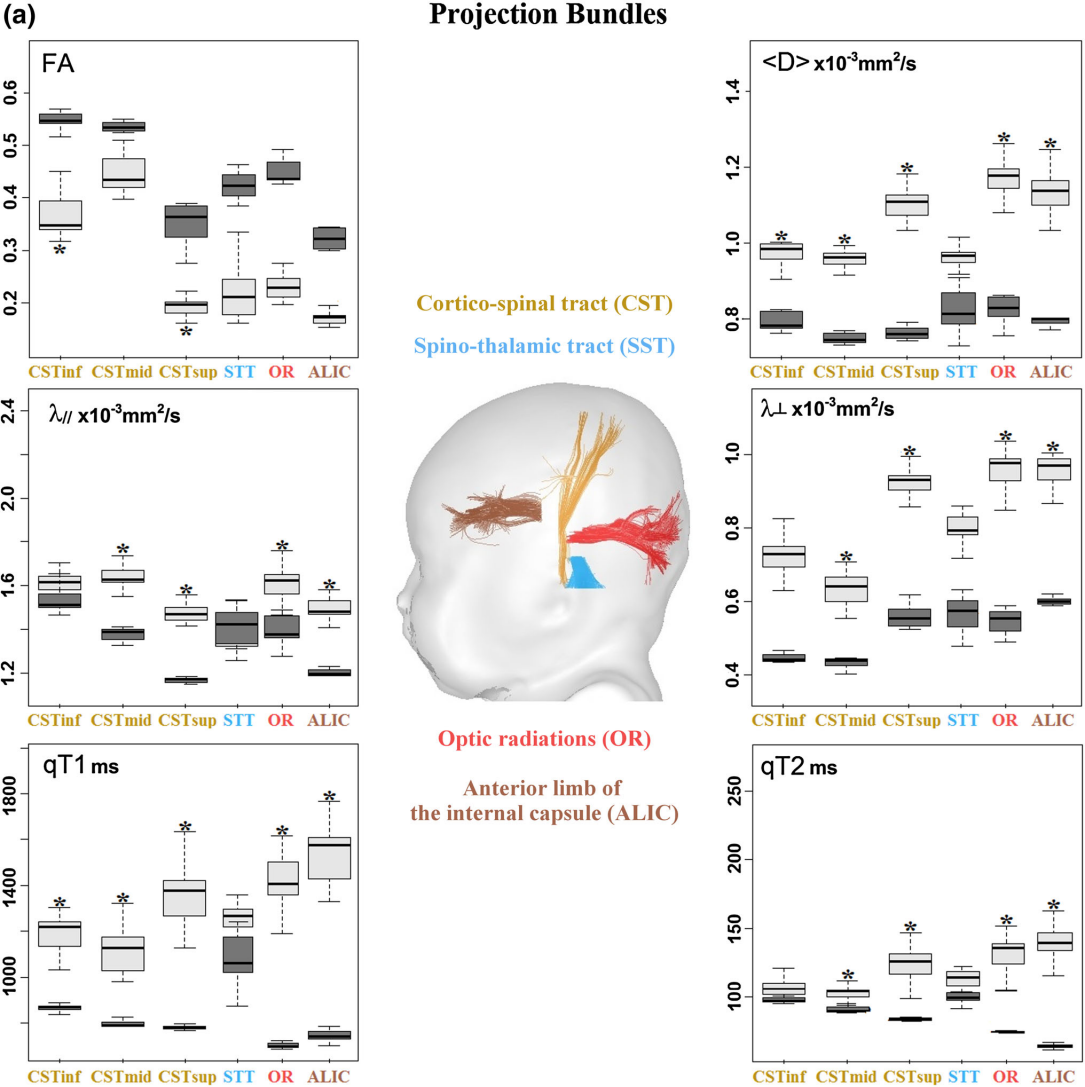

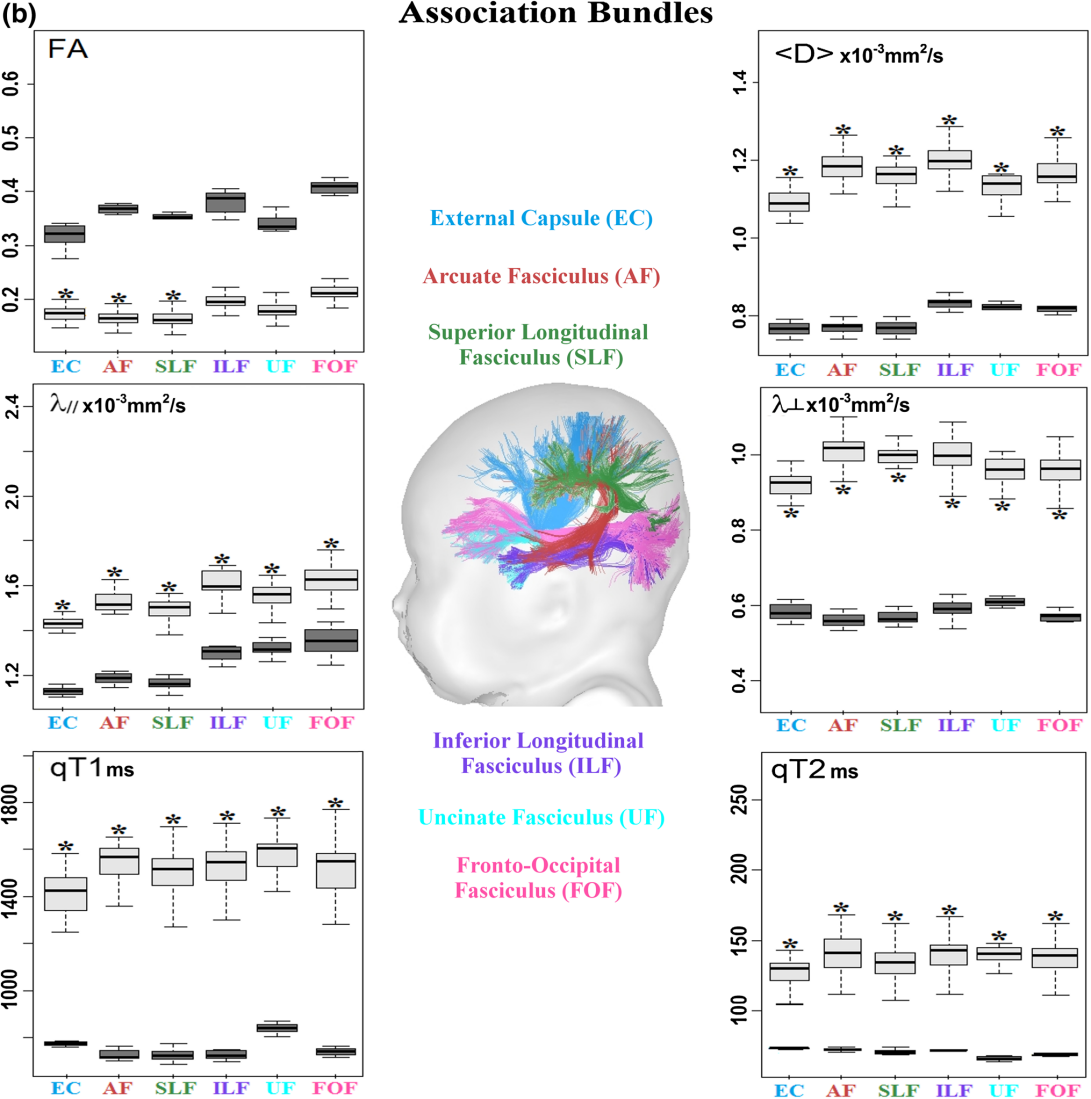

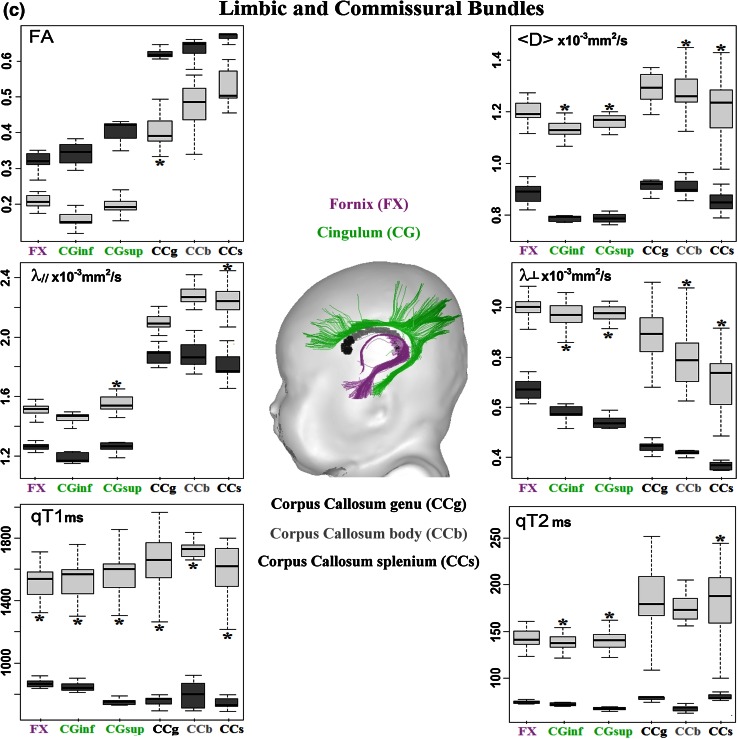
Quantification of the MRI parameters over the infant and adult groups. Mean and standard deviations of the parameters are shown across the bundles in the infant (*light boxes*) and adult (*dark boxes*) groups. *Asterisk* indicates that variations in the infant group could be attributed to the age-related changes by performing linear regressions with age (*R* ^2^ > 0.46, *p* < 0.05)

After correction of artifacts from motion and eddy currents (Dubois et al. 2014b), quantitative MRI and DTI maps were generated for all parameters (qT1, qT2, FA, <D>, λ_⊥,_ λ_║_) using Connectomist software (Fig. 1) (Duclap et al. 2012; Poupon et al. 2010). Whole brain tractography was performed according to a 4-order analytical Q-ball model and using regularized 3D tractography (Perrin et al. 2005). White matter bundles were identified in each subject using manually delineated regions of selection and exclusion (Huang et al. 2004). We selected 18 bundles that mature at different times and rates (Fig. 2) (Dubois et al. 2008):

- projection bundles: cortico-spinal tract CST with three subdivisions (inferior portion below the internal capsule, middle portion below the low centrum semiovale and superior portion), spino-thalamic tract STT, optic radiations OR, anterior limb of the internal capsule ALIC;
- association bundles: external capsule EC, arcuate fasciculus AF, superior SLF and inferior ILF longitudinal fascicles, uncinate fasciculus UF, fronto-occipital fasciculus FOF;
- limbic bundles: fornix FX, inferior CGinf and superior CGsup parts of the cingulum;
- commissural bundles: genu CCg, body CCb and splenium CCs of the corpus callosum.

For each subject, MRI parameters were quantified and averaged over the bundle length, taking into account fiber density (Dubois et al. 2006). All infant and adult values were further normalized by the corresponding means from the adult group.

### Implementation of the Mahalanobis approach

For all bundles, comparison of the normalized parameters in the infant and adult groups was performed using Mahalanobis distance $M$ (Mahalanobis 1936) as it allows taking into account the inter-subject variability and the parameters correlations in the adult group as well as their variability across the bundles:

1 $$M^{2} \left( {\vec{x}} \right) = \left( {\vec{x} - \vec{\mu }} \right)^{T} \sum\nolimits_{}^{- 1} \left( {\vec{x} - \vec{\mu }} \right),$$

where $\vec{x}$ is a multivariate vector describing an infant bundle, $\vec{\mu } = [1,1, \ldots ,1]$ is the mean vector for the corresponding bundle in the adult group and $\sum$ is a covariation matrix for parameters in adults. The smaller this distance, the closer the infant bundle to its mature adult stage. Mahalanobis distance can be equally calculated using the eigen systems representation:

2 $$M^{2} \left( {\vec{x}} \right) = \sum\nolimits_{i}^{n} {\left( {\left( {\vec{x} - \vec{\mu }} \right)\overrightarrow {{v_{i} }} } \right)^{2} /\lambda_{i} } ,$$

where $\overrightarrow {{v_{i} }}$ and $\lambda_{i}$ are the *n* eigenvectors and eigenvalues of the covariation matrix $\sum$. In our study Mahalanobis distance was calculated using four “independent” parameters: qT1, qT2, λ_⊥_, λ_║_ (FA and <D> were not included as they can be viewed as the functions of λ_⊥_, λ_║_).

Possible bias from non-dominant components that appears in small samples was compensated by substituting the smaller eigenvalues with the maximal eigenvalue (Takeshita et al. 1993):

3 $$M^{2} \left( {\vec{x}} \right) = \sum\nolimits_{i=1}^{4} {\left( {\left( {\vec{x} - \vec{\mu }} \right)\overrightarrow {{v_{i} }} } \right)^{2} /{ \hbox{max} }\left( {\lambda_{ 1} ,\lambda_{ 2} ,\lambda_{ 3} ,\lambda_{ 4} } \right)}$$

The age-related decrease in Mahalanobis distance was assessed for each bundle using linear regressions.

### Estimation of the calculation errors

Using formula (3) may lead to underestimation of the Mahalanobis distance because (1) the smaller eigenvalues are replaced by the maximal eigenvalue and (2) in small samples dominant components (components corresponding to bigger eigenvalues) tend to be slightly smaller than their true values (Takeshita et al. 1993). To take this into account, we estimated, for each bundle independently, average calculation errors for Mahalanobis distances between infants and adults. This estimation was performed using a computer simulation that compared Mahalanobis distances, calculated using 13 multivariate vectors randomly selected from the “true” distribution of the adult parameters across all bundles, with the “true” distances. The “true” distribution of the parameters was a Gaussian mixture distribution with the mean vector $\vec{\mu }_{\text{sim}} = [1;1;1;1]$ and the covariation matrix $\sum_{\text{sim}}$ determined from all 13 adults across all the bundles. A random sample of 13 vectors was taken from that distribution to estimate the Mahalanobis distance using (3) in each infant and for each bundle. These distances were compared with the “true” distances calculated using (1) and the “true” covariation matrix $\sum_{\text{sim}}$. The described procedure was repeated 1.000.000 times and the average positive $\sigma_{ + }^{ 2}$ and negative $\sigma_{ - }^{2}$squared normalized deviations between estimated and “true” distances were computed for each bundle independently.

### Comparison of the bundles maturation

In the group of infants with different ages, age-related changes of the Mahalanobis distance defined a maturational trajectory $M(b ; {\text{age}})$ for each bundle$b$, and comparing the maturation of two bundles $b_{i}$ and $b_{j}$ was equivalent to comparing the trajectories $M(b_{i} ; {\text{age}})$ and $M(b_{j} ; {\text{age}})$ across ages. To compare these trajectories at a given age, we considered the overlap between the two intervals $\left[ {M(b_{i} ;{\text{age}}) - \sigma_{ + } (b_{i} ;{\text{age}}) ; M(b_{i} ;{\text{age}}) + \sigma_{ - } (b_{i} ;{\text{age}})} \right]$ and $\left[ {M(b_{j} ;{\text{age}}) - \sigma_{ + } (b_{j} ;{\text{age}}) ; M(b_{j} ;{\text{age}}) + \sigma_{ - } (b_{j} ;{\text{age}})} \right]$. If these intervals overlapped, then the difference between $M(b_{i} ;{\text{age}})$ and $M(b_{j} ;{\text{age}})$ was set to zero and the two bundles were not distinguished one from another at this age. If the intervals did not overlap, the difference between $M(b_{i} ;{\text{age}})$ and $M(b_{j} ;{\text{age}})$ was equal to the smallest distance between the points belonging to the intervals, taken with a positive sign if $M(b_{i} ;{\text{age}}) < M(b_{j} ;{\text{age}})$ ($b_{i}$ was more mature than $b_{j}$ at this age) and with a negative sign in the opposite case.

To compare two bundles across the whole age range, these differences were considered between the corresponding age-points on their maturational trajectories. If these differences were significantly different from zero (paired *t* test over the infant group) then the bundles were said to have different maturational trajectories. The result of the pair-wise comparisons between all bundles created a partial maturational order on the set of bundles that was presented as a graph, showing complex maturational relationships. Statistical tests were considered with a 0.95 significance level, corrected for multiple comparisons using FDR approach. Relationships that failed to reach the 0.95 significance level were also tested at the level of 0.9.

### Comparison of the Mahalanobis approach with univariate approaches

As for univariate approaches, we evaluated the variations with age of each normalized MRI parameter, including FA and <D>, for each of the bundles. Similarly to the Mahalanobis distance approach, partial ordering of the bundles was performed using each MRI parameter independently. All partial orders (from Mahalanobis distance and from each parameter) were compared in terms of (1) the number of discriminated relationships between the bundles; (2) presence of violations in five a priori known maturational relationships: spino-thalamic tract, cortico-spinal tract and optic radiations should be among the most fast-maturing bundles, while anterior limb of the internal capsule and arcuate fasciculus should be among the most slowly maturing (Yakovlev and Lecours 1967; Paus et al. 1999; Zhang et al. 2007).

Additionally, we evaluated which strategy made better predictions on the maturational age using a “leave-one-out” approach. Because of the short age range, changes in the normalized MRI parameters and in the Mahalanobis distance with age were fitted with linear equations (where appropriate with *R* ^2^ > 0.46 corresponding to *p* < 0.05). To make predictions for each bundle, the fitting was done using all but one infant, and his/her age predicted by the fitting was then compared with the real age. The described procedure was repeated for all infants and the prediction errors were averaged.

### Implementation of a general equation of the maturation

As detailed in the results section, the derivative $- \frac{{dM(b; {\text{age}})}}{{d{\text{age}}}}$ was found to linearly depend on the average (over the age range) Mahalanobis distance $\left\langle {M(b)} \right\rangle$, suggesting that the “general maturational equation” should take the exponential form:

4 $$M\left( {b;{\text{age}}} \right) = a\left( b \right) \times { \exp }( - c \times {\text{age}})$$

5 $${\text{or}}:M\left( {b;{\text{age}}} \right) = A_{0} \times { \exp }\left( { - c \times \left( {{\text{age}} - {\text{age}}_{0} \left( b \right)} \right)} \right),$$

where $A_{0} ,c$ are constants and ${\text{age}}_{0} (b)$ can be interpreted as the age of the maturation onset for a bundle $b$. This description further enabled to compute a relative maturational delay between two bundles $b_{i}$ and $b_{j}$:

6 $${\text{age}}_{ 0} \left( {b_{i} } \right) - {\text{age}}_{0} \left( {b_{j} } \right) = \frac{1}{c} \times \ln \left( {a\left( {b_{i} } \right)/a\left( {b_{j} } \right)} \right)$$

When bundle groups were defined in the Mahalanobis ordering, we indicated the minimal and maximal delays between bundles.

To investigate whether this exponential model remains adequate at older developmental stages, it was tested on a 34-week-old infant. For all bundles, the “true” Mahalanobis distances were calculated according to the infant’s data and Eq. 3, and compared with the values predicted by the exponential Eq. 4.

## Results

### Changes in the normalized MRI parameters and Mahalanobis distance with age

Despite low brain maturation in infants, we obtained high-quality MRI maps in all subjects (Fig. 1), as well as reliable bundle reconstructions and parameter quantification for all bundles (Fig. 2). In all infant bundles, fractional anisotropy was lower than in adults, while other parameters (relaxation times and diffusivities) were higher. Besides, the means and the variabilities of the infant parameters were not the same across the bundles (Fig. 2), reflecting differences in the maturational stages and in the rates of the maturational changes over the age range. The global picture was even more complex because of unequal mean values and unequal variability of the parameters across the bundles in the adult group. This confirmed the need for normalization of the parameters by the corresponding means over the adult group in order to reliably compare the infant and adult groups and to highlight maturational differences across the bundles.

Over this short developmental period, normalized parameters changed with age (increase in fractional anisotropy, decrease in other parameters). For each parameter, the observed differences across the bundles suggested that certain bundles (e.g. spino-thalamic and cortico-spinal tracts) matured faster than the others; however, the majority of the bundles could not be differentiated one from another.

**Fig. 3:**
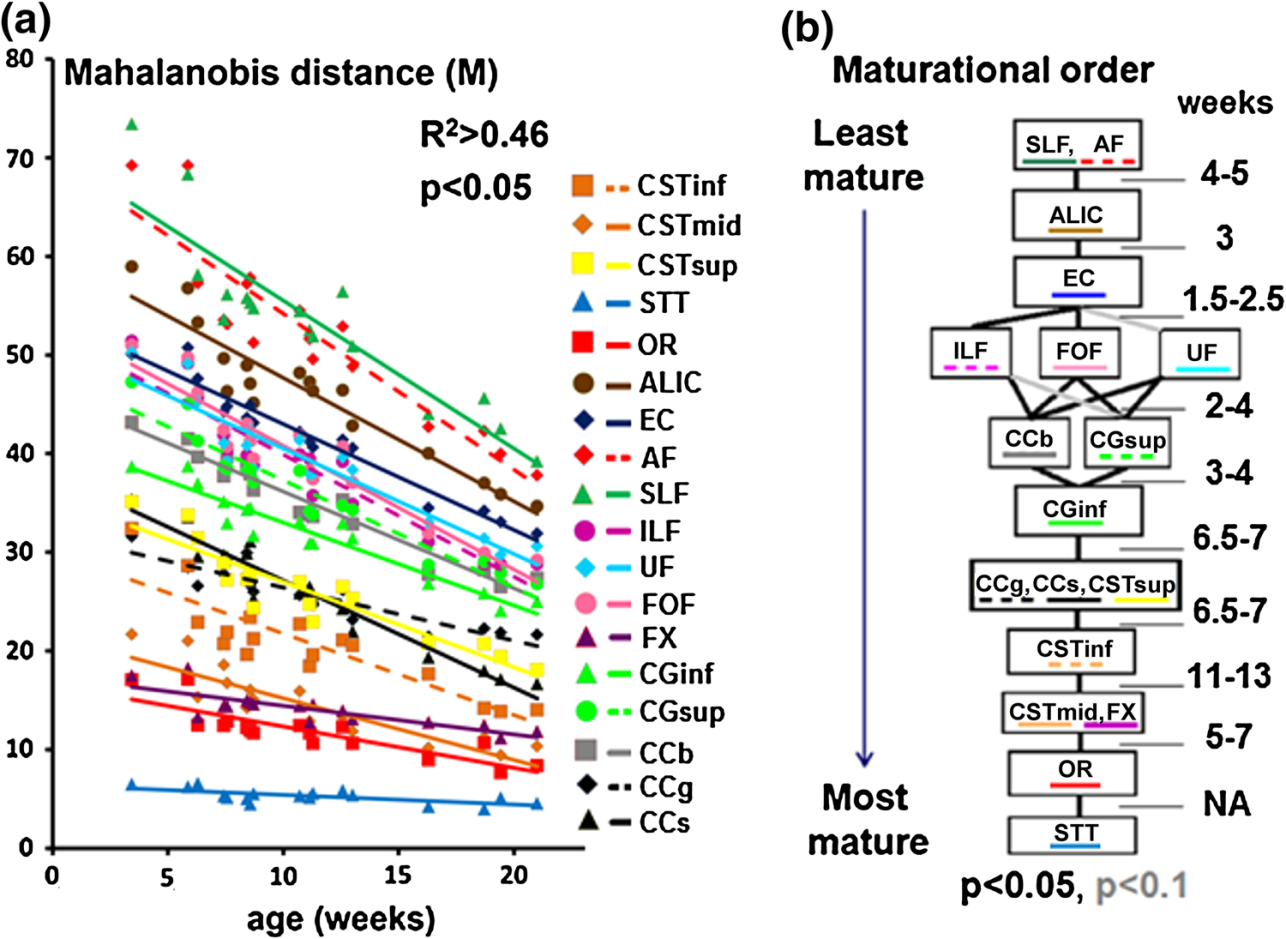
Bundle maturational order revealed by the Mahalanobis distance. **a** Mahalanobis distances to the adult stage progressively decreased with the infants’ age in all bundles and were modeled by linear fitting over this short developmental period. The rate of decrease was slower in the bundles already advanced in maturation (smaller distances) than in those showing higher distances to the adult bundles (see Fig. 4). **b** Maturational relationships between the bundles are represented as a graph. Bundles showing advanced maturation are close to the *bottom*; those with delayed maturation are on the *top*. *Gray lines* (between CGsup and ILF; UF and EC) mark relationships that failed to reach statistical significance (0.05 < *p* < 0.1). Relative maturational delays (in weeks) between the bundles or bundle groups are indicated on the *right side*. Delays between the spino-thalamic tract (STT) and other bundles were not considered (see text for explanations). See Fig. 2 for abbreviations

Besides, Mahalanobis distance was computed for each bundle in all infants: it decreased with age in all bundles, reflecting bundles’ maturation (Fig. 3a). It seemed to provide better discrimination of the bundles than other MRI parameters, confirming the spino-thalamic and cortico-spinal tracts to be among the most mature bundles. Contrarily to univariate parameters (Fig. 2), age-related linear regressions were significant for all bundles (Fig. 3a). Despite the relatively small size of the adult group, simulations showed that Mahalanobis distance was calculated with an acceptable precision (Online Resource 1), with average (over all bundles) positive and negative deviations from the true values being equal to 1.0 ± 0.3 % and 6.0 ± 1.8 %, respectively.

### Ordering the bundles maturational trajectories with Mahalanobis approach

Pair-wise comparisons of Mahalanobis distances across the bundles created a partial maturational order represented as a graph (Fig. 3b). As expected, the most mature were the bundles responsible for sensory and motor functioning: spino-thalamic tract was the most advanced followed by optic radiations and the cortico-spinal tract; thus, most projection bundles (except the anterior limb of the internal capsule) appeared more mature than limbic, commissural and association bundles. The middle portion of the cortico-spinal tract was advanced relatively to its inferior and superior parts. As for limbic bundles, the fornix was more mature than the cingulum. The splenium and genu of the corpus callosum were more mature than the body. Concurrently and consistently with our expectations, the most delayed maturation was observed in the arcuate and superior longitudinal fasciculi and in the anterior limb of the internal capsule. Some bundles were grouped together when the comparison did not reveal any significant differences in their maturational trajectories: for example, the genu and splenium of the corpus callosum and the superior portion of the cortico-spinal tract. The obtained ordering did not violate the a priori known relationships and revealed 142 out of 153 (17 infants × 18 bundles divided by 2) maximal possible relationships.

### Comparison of the Mahalanobis approach with univariate approaches

Maturational orderings were also obtained according to each normalized MRI parameter and compared with the Mahalanobis approach. For all univariate parameters, the number of discriminated relationships was below 90, and for all of them, except longitudinal diffusivity, maturational orders contained violations of the a priori known relationships (Table 1). The most common violation concerned the placement of the optic radiations relatively to other bundles in the maturational order: they were classified as relatively immature and placed at the same level as either the arcuate fasciculus (for qT2) or the anterior limb of the internal capsule (for qT1, <D>, FA, λ_⊥_). Thus, according to our comparison criteria, Mahalanobis distance showed the best performance (Table 1). Note that none of the 11 relationships unrevealed by the Mahalanobis distance was discriminated by any of the univariate approaches.

**Table 1:** Comparison of the Mahalanobis distance approach (*M*) with other univariate approaches

	*n*	violations	Prediction errors (%)
M	142	–	17 ± 8
FA	74	1. Spino-thalamic tract was among the least mature bundles.	46 ± 20
		2. Optic radiations and anterior limb of the internal capsule were at the same immature level.	
<D>	72	Optic radiations and anterior limb of the internal capsule were placed at the same intermediate maturational level.	45 ± 24
λ_║_	76	–	54 ± 38
λ_⊥_	70	1. Optic radiations were less advanced in maturation than anterior limb of the internal capsule.	44 ± 21
		2. Cortico-spinal tract and anterior limb of the internal capsule were at the same maturational level.	
qT1	90	Optic radiations were among the least mature bundles.	27 ± 20
qT2	89	Optic radiations and arcuate fasciculus were placed at the same intermediate maturational level	21 ± 10

Mahalanobis distance was able to discriminate more maturational relationships between the bundles (*n* out of 153) than other univariate approaches and it did not violate a priori known maturational relationships (violations). Additionally, prediction errors (in %) of the maturational age in the leave-one-out validation were smaller for Mahalanobis distance approach than for other univariate approaches (for details see Online Resource 2)

Additionally, leave-one-out validations confirmed that linear models based on the Mahalanobis distance provided better predictions of the maturational age than univariate approaches in 14 out of 18 bundles (Table 1, Online Resource 2). Prediction errors for Mahalanobis distance were of 17 ± 8 % on average over all bundles. These errors were higher for all other parameters (Table 1, Online Resource 2).

### General equation of the maturation according to the Mahalanobis distance

**Fig. 4:**
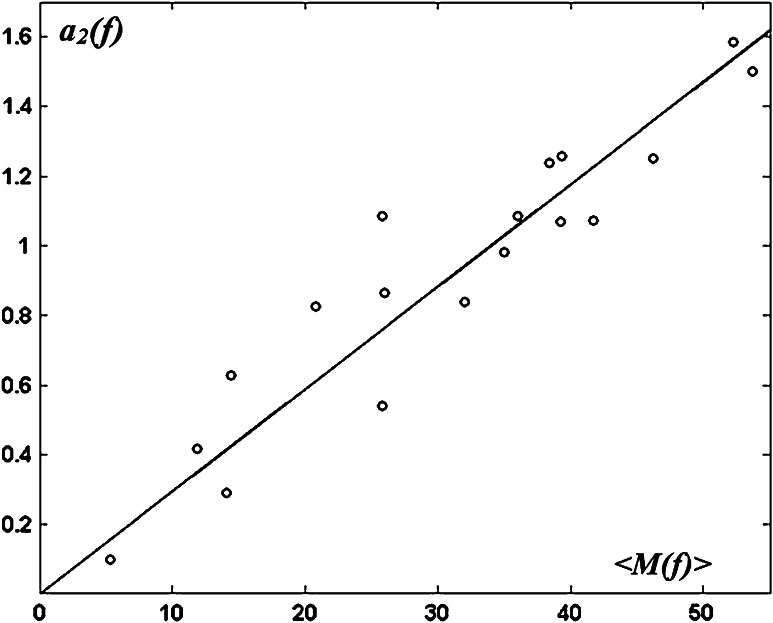
Relationship between the speed of changes of the Mahalanobis distance and the maturational stage. For each bundle b, the age-related decrease in the Mahalanobis distance was modeled by a linear approximation: $M\left( {b;age} \right) = a_{1} \left( b \right) - a_{2} \left( b \right)*age$. Across the bundles, the corresponding slopes $(a_{2} \left( b \right))$ linearly increased with the mean Mahalanobis distances $\left\langle {M(b)} \right\rangle$ (*R* ^2^ = 0.89)

Considering linear approximations of the maturational trajectories with age $M\left( {b;{\text{age}}} \right) = a_{1} \left( b \right) - a_{2} \left( b \right)*{\text{age}}$, we found that for all bundles, the slope $a_{2} \left( b \right)$ (or $- \frac{{dM\left( {b; {\text{age}}} \right)}}{{d{\text{age}}}}$) linearly depended on the average Mahalanobis distance $\left\langle {M(b)} \right\rangle$ (over the age range) (Fig. 4, *R* ^2^ = 0.89). Thus, the maturational trajectories were further modeled by exponential decays (Eq. 4). Fitting our data with Eq. 4 resulted in constant $c$ = 0.03075 and the bundle-related coefficients $a(b)$ detailed in Table 2.

**Table 2:** Exponential fitting of the Mahalanobis distance (Eq. 4) for different bundles

	CSTinf	CSTmid	CSTsup	STT	OR	ALIC
*a*(*b*)	29.2	20.4	36.4	7.3	16.6	64.4

	EC	AF	SLF	ILF	UF	FOF
*a*(*b*)	58.1	73.1	74.9	53.7	54.7	55.0

	FX	CGinf	CGsup	CCg	CCb	CCs
*a*(*b*)	19.5	44.6	50.3	35.8	48.8	36.4

Bundle-related coefficients $a\left( b \right)$ are specified here: they were further used for calculation of the relative maturational delays between the bundles or bundle groups (Eq. 6). See Fig. 2 for abbreviations

The relative maturational delays between the bundles were further computed using Eq. 6 (see results in Fig. 4b). The minimal delay was 1.5 weeks between the fronto-occipital fasciculus and the external capsule, and the maximal delay was 13 weeks between the fornix and the inferior portion of the cortico-spinal tract. Delays between the spino-thalamic tract and other bundles were not considered because for this tract none of the individual parameters was able to reveal any significant age-related changes: thus, the Mahalanobis distance could not make use of any of them, having an artificially flat slope in the age-related changes, which resulted in overestimations of the relative maturational delays between the spino-thalamic tract and other bundles. The total delay between the optic radiations and the least mature group (arcuate and superior longitudinal fasciculi) was estimated to be 48–49 weeks.

Finally, this exponential model was tested on a 34-week-old infant to investigate its performance for older ages. Comparison of true and predicted values suggested a good agreement with an average prediction error of 13.5 % (Table 3). Prediction errors tended to be smaller for bundles with delayed maturation (e.g. 3 and 4 % for uncinate and arcuate fasciculi, respectively) than for bundles with advanced maturation (e.g. up to 33 % for spino-thalamic tract and the middle portion of the cortico-spinal tract), presumably because greater changes in the Mahalanobis distance (corresponding to bundles with delayed maturation) could be better approximated over that age range than smaller changes (corresponding to bundles with advanced maturation). However, certain bundles did not follow this rule: although optic radiations were among the most advanced in maturation, the prediction error for them was surprisingly low (1 %).

**Table 3:** Evaluation of the maturational model in a 34-week-old infant

	CSTinf	CSTmid	CSTsup	STT	OR	ALIC
Predicted	10.3	7.2	12.8	2.6	5.8	22.6
True	9.8	5.4	11.6	3.9	5.7	24.8
Error (%)	5	33	11	33	1	9

	EC	AF	SLF	ILF	UF	FOF
Predicted	20.4	25.7	26.3	18.9	19.2	19.3
True	18.3	24.6	26.4	17.4	19.8	18.7
Error (%)	12	4	0.4	8.6	3	3

	FX	CGinf	CGsup	CCg	CCb	CCs
Predicted	6.9	15.7	17.7	12.6	17.1	12.8
True	7.6	15.3	17.7	11.8	14.5	11.6
Error (%)	9	2	0.2	7	18	10

For each bundle, the value of the Mahalanobis distance predicted by the maturational model (Eq. 4) and the true value calculated using Eq. 3, are detailed. The average prediction error across the bundles was 13.5 %. See Fig. 2 for abbreviations

## Discussion

In this article we have proposed an original multi-parametric approach for quantitative in vivo evaluation of white matter maturation. This approach enabled demonstrating the asynchrony in the bundles’ maturation more reliably than conventional univariate approaches within the period from 3 to 21 weeks of post-natal age. It further suggested a general quantitative description of the maturation that enabled estimating the relative maturational delays between the bundles.

### Multi-parametric vs. univariate approaches

MRI and DTI parameters provide exquisite details on white matter maturation, and their age-related changes are known to reflect undergoing maturational processes. However, none of these parameters alone can describe the complexity of white matter maturation because different MRI/DTI parameters are sensitive to different tissue properties and thus, to different stages of the maturational processes (Dubois et al. 2008; Steen et al. 1997; Engelbrecht et al. 1998; Barkovich et al. 1988; Dubois et al. 2014a). To overcome this difficulty, multi-parametric models that take advantage of the complementary dependencies of the MRI parameters on maturational processes should come on stage (Prastawa et al. 2010; Sadeghi et al. 2013; Vardhan et al. 2012). Such models should provide a measure of a maturational distance between infant and adult brains. They should also take into account variations and covariations of the parameters across different bundles in the adult group because a difference between infant and adult parameters is relevant only if it is superior to the normal variations within the adult group.

To our knowledge, there is only a couple of recent studies trying to combine both MRI and DTI parameters in a single maturational model (Sadeghi et al. 2013; Prastawa et al. 2010). Sadeghi et al. (2013) used Gompertz functions to model age-related changes in both FA and the intensities of T1- and T2-weighted images. Prastawa et al. (2010) suggested a non-linear growth model based on modified Legendre polynomial basis, that was used to create maturation maps, using five modalities: longitudinal and transverse diffusivities, proton density and intensity of T1- and T2-weighted images. Instead of quantitative T1 and T2 relaxation times, both of these studies used the intensities of T1- and T2-weighted images, which are hardly comparable across brain regions and across subjects because of signal inhomogeneities and of varying acquisition tunings. Furthermore, none of these models took into account the differences in the parameters and their variations at the mature adult stage. Finally, these studies provided region-specific rather than tract-based information possibly mixing the information about different bundles passing at the same location.

Our approach is free from these drawbacks, and to our knowledge, it is the first study using Mahalanobis distance to evaluate brain maturation. Mahalanobis, rather than Euclidean distance, was chosen because MRI parameters are correlated and cannot be viewed as completely independent variables. Moreover, their covariation matrices and thus, the eigensystems are different across bundles. In our study, Mahalanobis distance was calculated using four parameters: quantitative relaxation times (qT1, qT2), transverse (λ_⊥_) and longitudinal (λ_║_) diffusivities. Fractional anisotropy (FA) and mean diffusivity (<D>) were not considered because they can be viewed as functions of λ_⊥_ and λ_║_, and including them may result in a degenerate covariation matrix.

As for the approach validation, it outperformed univariate approaches in bundle discrimination at different maturational stages, and its discrimination capacity was extremely high. Our approach suggested a more reliable ordering of the bundles according to their relative maturation and showed smaller prediction errors of the maturational age.

### Mapping the asynchrony of the white matter maturation

Although this study presents a preliminary investigation based on a small number of subjects, the proposed multi-parametric approach enabled precise and reliable demonstration of the asynchrony in the bundles maturation in the infant brain. The suggested maturational order was in good agreement with *post-mortem* studies (Yakovlev and Lecours 1967; Flechsig 1920), confirming maturation of the sensory and motor pathways before association bundles. The spino-thalamic tract was the most advanced in maturation, followed by the optic radiations, the middle portion of the cortico-spinal tract and the fornix. Most projection bundles (except the anterior limb of the internal capsule) thus appeared more mature than limbic, commissural and association bundles. As for limbic and commissural bundles, the fornix was more mature than the cingulum, and the splenium and genu of the corpus callosum were more mature than the body. The bundles with most delayed maturation included the arcuate and superior longitudinal fasciculi, the anterior limb of the internal capsule and the external capsule.

With this approach, the middle portion of the cortico-spinal tract was more advanced in maturation relatively to its inferior and superior parts, in agreement with previous in vivo imaging studies showing that central regions mature before peripheral regions (Prastawa et al. 2010; Gao et al. 2009). Nevertheless, earlier maturation of the middle portion in comparison with the inferior portion may seem to contradict the known rule of the caudo-cephalic direction of the myelin progression (Yakovlev and Lecours 1967; Flechsig 1920). However, one should keep in mind that the actual myelination sequence is very complex, being also governed by several other rules and showing multiple exceptions (van der Knaap et al. 1995; Kinney et al. 1988; Flechsig 1920). Here our observations in the cortico-spinal tract may have several explanations. First, this tract includes both sensory (thalamo-cortical) and motor (cortico-spinal) fibers, which are not supposed to myelinate with the same sequence and topography since most tracts become myelinated in the direction of the impulse conduction (van der Knaap et al. 1995). Furthermore, it may simply reflect the fact that the posterior limb of the internal capsule, which here corresponds to the delimitation between the inferior and middle portions, already shows the presence of myelin at term and undergoes very rapid myelination (Kinney et al. 1988; Flechsig 1920), being one of the first to get the “myelinated” appearance on T1- and T2-weighted images in term newborns (Paus et al. 2001; Rutherford 2002), probably due to a high compactness of the fibers. Second, as remarked by Kinney et al. (1988), early myelination onset does not predict early myelin maturation. For example, optic radiations, unlike cortico-spinal tract, do not show evidence of myelin at term, but nevertheless get faster to the mature stage (Kinney et al. 1988). As myelination of the cortico-spinal tract is not restricted to the considered short developmental period (3–21 weeks) but continues up to 142 weeks (Kinney et al. 1988), it could have happened that the most dynamic changes during this period were in the middle portion, making our approach classify it as relatively more advanced. Similarly, although corpus callosum starts to myelinate after the cortico-spinal tract, it gets to the mature stage much faster than the superior portion of the cortico-spinal tract in the corona radiata (Kinney et al. 1988). As myelination of both of these bundles is not restricted to the first post-natal months, it is possible that during this period these bundles were at the same maturational stage, and thus grouped together.

Next, we should also highlight that the Mahalanobis distance is not directly linked to the myelin content but rather reflects the whole ensemble of various maturational processes underlying age-related changes in the MRI/DTI parameters used for its calculation (qT1, qT2, λ_║_ and λ_⊥_). Besides, it might also be possible that co-registration of the different imaging modalities (qT1, qT2, DTI) in the lower parts of the brain was not as perfect as in the central regions (because geometric distortions related to EPI sequences are more prominent in the brainstem) and the relative maturational degree was slightly underestimated in the inferior portion of the cortico-spinal tract.

On the other hand, the bundles that were grouped together should not be considered as bundles with identical maturation, but rather as bundles for which maturational relationships could not be revealed using the proposed approach and the available data. Indeed, the middle portion of the cortico-spinal tract and the fornix were grouped together, whereas the fornix matures somewhat later than this projection tract (Yakovlev and Lecours 1967). Such unrevealed relationships may stem from a high inter-subject variability relatively to the age-related changes: for example, for the fornix, only Mahalanobis distance and qT1 showed significant age-related changes, nevertheless leading to high prediction errors in the leave-one-out validation after regressing out the age-related effects. Increasing the number of subjects may possibly help to further improve the discrimination capacity of our approach, as discussed below. Nevertheless, one should notice that none of such unrevealed relationships between the bundles could be discriminated by any of the univariate approaches. Another explanation could be that neither Mahalanobis distance nor individual MRI parameters directly reflect brain myelination, being influenced by all kinds of undergoing maturational processes that overlap in time (Dubois et al. 2014a). Thus, in future studies it will be of interest to compare our model to a novel MRI parameter, named myelin water fraction (MWF) (Deoni et al. 2012), which is supposed to be more directly linked to the myelin content (see discussion below) and may help to discriminate the unrevealed relationships between the bundles (Kulikova et al. 2014).

With all these considerations in mind, one should remember that there is still no gold standard for the in vivo evaluation of the white matter maturation and thus, direct comparison of our results and other studies should be made with caution. *Post-mortem* studies (Yakovlev and Lecours 1967; Flechsig 1920) provide region-specific but not bundle-specific information and thus, may mix up information about various bundles that pass at the same location. As for in vivo studies, there were only a few attempts to give a precise definition of a bundle maturational stage using multi-parametric MRI data. The model of Dubois et al. (2008) was based on the estimation of the global bundle maturation by progression through four stages, which took into account both the maturation state and speed of each bundle, calculated from DTI indices (mean diffusivity and fractional anisotropy), in comparison with the average over all bundles and according to age. This model suggested that the cortico-spinal tract appeared the most mature, followed by the spino-thalamic tract and the fornix, then the optic radiations, the arcuate and inferior longitudinal fasciculi, and the least mature were the anterior limb of the internal capsule and the cingulum. However, the model was not supported in three bundles (corpus callosum, external capsule, and uncinate fasciculus) and did not give quantitative assessment of the relative maturational delays between the bundles.

Prastawa et al. (2010) calculated an absolute maturational measure from the total growth rate for a set of multimodal observations (longitudinal and transverse diffusivities, proton density and intensity of T1- and T2-weighted images). The relative maturational measure was calculated as the time shift required to transform a maturational curve for a given bundle to a reference curve computed from the posterior limb of the internal capsule (because of its known early myelination). This model confirmed the known temporal order of the white matter maturation: (1) brain regions related to basic functions such as sensory and motor information processing are the most advanced in the maturation; (2) central regions of the white matter tracts mature before peripheral sub-cortical regions. However, this study did not report any quantitative results on the relative maturational delays between white matter regions, and it was focused on different regions rather than on different bundles.

Vardhan et al. (2012) proposed using the Hellinger distance to measure age-related changes in the intensities of T1- and T2-weighted images. This strategy also demonstrated that maturation begins in posterior regions and that frontal regions mature later on. The authors further confirmed that T1 and T2 modalities are likely to reflect different maturational properties, as revealed by a time lag in the changes of T2-weighted contrast compared with T1-weigthed images. However, this study was again region- and not bundle-specific, and used weighted rather than quantitative images.

Other studies on white matter maturation used predominantly univariate approaches, trying to classify the bundles based on changes in a single modality: for example, fractional anisotropy (Imperati et al. 2011), quantitative qT1 (Steen et al. 1997) or qT2 (Engelbrecht et al. 1998) relaxation times, etc. Although these studies were able to capture the general pattern of white matter maturation, the exact placement of bundles in the maturational order may be biased because none of the MRI parameters alone can explain the whole ensemble of processes underlying maturation.

Fitting the data with our model further suggested that different white matter bundles follow a similar maturational trajectory but with different developmental onsets. This finding, being in agreement with (Prastawa et al. 2010), allowed us deriving a “general” maturational equation: similarly to univariate studies during childhood over a larger age range (Watanabe et al. 2013; Engelbrecht et al. 1998; van Buchem et al. 2001; Lebel et al. 2012), changes in the Mahalanobis distance with age in infants could be described by an exponential decay. This modeling allowed us to compute the relative maturational delays between the bundles, confirming that the most dramatic changes in the white matter occur during the first post-natal year, with a total relative maturational delay of 49 weeks between the most and the least mature bundles. That is why we tested our model on an older infant, with fair predictions for almost all bundles. The model tended to be less accurate for bundles with advanced maturation in which inter-subject variability was likely to become comparable with age-related changes. Nevertheless, further studies with larger sample sizes may enable to clarify this issue.

### Technical considerations

When studying normal brain development, researchers always face the problem of data acquisition in healthy unsedated infants and children. To avoid devastating motion artifacts, data are usually acquired during natural sleep, trying to keep the acquisition sequences as short as possible. In our study we used EPI single-shot spin-echo sequences, which allowed us to acquire the whole multimodal dataset in less than 15 min. Although using these sequences may be complicated by image distortions, distortions for qT1, qT2 and DTI images were relatively coherent and did not pose problems for co-registration, except maybe in the brainstem as discussed above. Because the parameters were quantified over the bundles, our analysis was less affected than voxel-by-voxel analyses, and distortions were most prominent in the frontal regions, which lay apart from the majority of the bundles analyzed in our study.

Comparison of the parameters averaged over different bundles allowed us to capture the general picture of the maturational asynchrony. Although voxel-by-voxel analysis may potentially reveal more details on local maturational changes, it would require exact correspondence between cerebral structures among individuals and thus, precise co-registration between infant and adult images, which remains hardly achievable because babies’ and adults’ brains are not homothetic due to asynchronous growth of cerebral regions. Furthermore, as maturation is not homogeneous along axons and bundles (McArdle et al. 1987; McCart and Henry 1994), it would be interesting in future studies to split all bundles into several parts (as it was done here for the cortico-spinal tract, the cingulum and the corpus callosum) and to analyze them separately. In the same way, analyzing separately the left and right bundles would highlight inter-hemispherical asymmetries that may exist in bundles such as the arcuate fasciculus (Dubois et al. 2008; Lebel and Beaulieu 2009).

In our study the bundles were reconstructed using manually delineated regions of selection and exclusion. To avoid inter-subject variability, these regions were delineated according to predefined rules (Catani et al. 2002; Dubois et al. 2006). Although in adult subjects white matter bundles can be extracted using multiple automatically placed regions-of-interest (Suarez et al. 2012) or pre-defined bundle atlases and clustering techniques (Guevara et al. 2010), such approaches may fail to reliably extract the bundles in infant datasets. To our knowledge, so far there are no approaches designed specifically for the age ranges considered in our study. Thus, to be coherent in terms of bundles identification between infant and adult subjects, bundles were reconstructed in the same way in both groups.

The number of infants (*N* = 17) included in this work may seem relatively low to derive definite conclusions about white matter maturation, particularly for the white matter bundles showing higher prediction errors in the leave-one-out validation (e.g. spino-thalamic tract, fornix), i.e. the bundles in which inter-subject variability in the MRI/DTI parameters or in the Mahalanobis distance was relatively high as compared to the age-related changes over the considered age period. Nevertheless, as most of the bundles indeed showed dramatic changes of both the Mahalanobis distance and the MRI/DTI parameters over this short developmental period (3–21 weeks), we were able to reveal a general scheme of the maturational asynchrony across the bundles, even in a cross-sectional analysis. However, the main goal of the present study was not to make definite conclusions about the exact bundle maturational order, but rather to introduce and explore the Mahalanobis distance approach and to demonstrate its advantages over conventional univariate approaches. Indeed, even in a small size group, Mahalanobis distance approach showed better performance than conventional approaches in bundle discrimination and suggested a more reliable bundle ordering with smaller prediction errors of the maturational age. Although the maturational order may be considered here as preliminary, requiring further validation in studies with larger sample sizes, notably to distinguish the bundles that were grouped together (see above), the obtained results suggest that our approach may be a promising candidate for the evaluation of pathological development or neuro-degeneration of the white matter when it is not possible to acquire large datasets. Similarly, interpolation of our model to older ages should be made with caution, since it was only tested in a single 34-week-old infant for demonstration purposes. Testing whether the exponential model and the Mahalanobis approach are indeed valid at older ages would require recruiting many healthy infants and toddlers during the second semester of the first post-natal year and the first semester of the second year (when, according to our model, the Mahalanobis distances in all bundles should decrease below the 10 % of their initial values). This is hardly achievable because it is exceptional to have healthy infants and toddlers spontaneously asleep (without sedation) during scanning at those ages.

The precision of our approach also depends on the size of the adult group, used to calculate covariation matrices of the MRI/DTI parameters. The exact relationship between the calculation errors of the Mahalanobis distance and the group size was described by Young (1978), and a number of strategies were introduced to compensate the bias in small samples (Iwamura et al. 2002; Jorgensen and Rothrock 2010; Omachi et al. 2000; Takeshita et al. 1993). In our study we applied the correction strategy suggested by Takeshita et al.(1993), and our computer simulations suggested that Mahalanobis distances were calculated with an acceptable precision that enabled to discriminate the maturational trajectories of different bundles.

Finally, another way to further improve the Mahalanobis approach may be to include other MRI-derived metrics, like myelin water fraction (MWF) (Deoni et al. 2012), magnetization transfer ratio (MTR)(van Buchem et al. 2001) or macromolecular tissue volume (MTV) (Mezer et al. 2013), that may yield additional information on maturational processes. MWF relies on the multi-compartment modeling of T1 and T2 relaxation signals and is thought to better correlate with the degree of bundle myelination than other MRI parameters. However, MWF calculation has still no gold standards and requires long acquisition and post-processing times (Deoni et al. 2013). MTR is another parameter sensitive to the myelin content, based on the exchange of magnetization between free protons and protons bounded to macromolecules, such as the cholesterol component of myelin, cerebrosides and phospholipids (Koenig 1991; Kucharczyk et al. 1994). Although MTR can be used to measure myelin content, it is also sensitive to multiple other factors (Nossin-Manor et al. 2013). Finally, MTV is a recent MRI parameter proposed by Mezer et al. (2013), which quantifies the non-water volume. Combining MTV with qT1 mapping may potentially provide new information about variations in local physico-chemical environments, while combining it with DTI imaging may help to distinguish between variations in tissue orientation and tissue density. Including these parameters into analysis will change the covariation matrices and likely result in different values of Mahalanobis distances, potentially increasing the discrimination capacity of the approach; however, we can expect the bundle maturational order to be preserved and the maturational model to remain exponential.

## Conclusion

Using Mahalanobis distance, computed from relaxation times and DTI diffusivities, has been shown relevant for in vivo evaluation of the white matter maturation in infants. It confirmed the known spatio-temporal sequence of the white matter maturation, showing the spino-thalamic tract, the optic radiations, the cortico-spinal tract and the fornix to be among the most fast maturating bundles, while the superior longitudinal and arcuate fasciculi, the anterior limb of the internal capsule and the external capsule had the most delayed maturation. Of importance, Mahalanobis distance could reveal more details on the maturational differences between the bundles and enabled more precise predictions of the maturational ages than conventional univariate approaches. Additionally, our approach suggested a maturational model that enabled calculating the relative maturational delays between the bundles and confirmed that the most dramatic maturational changes should occur during the first post-natal year. As the proposed approach is based on a short acquisition protocol and showed good performance even in a small-size group, it may be easily adapted to clinical studies when it is not possible to acquire large datasets (e.g. in rare diseases such as leukodystrophies) or when the patients cannot withstand long acquisitions (e.g. psychiatric patients).

## Electronic supplementary material

Below is the link to the electronic supplementary material.

Supplementary material 1 (PDF 126 kb)

Supplementary material 2 (PDF 167 kb)
