# Supplementary material for: Multi-parametric evaluation of the white matter maturation
Source: Brain Struct Funct. 2014 Sep 3;220(6):3657–72. doi: 10.1007/s00429-014-0881-y (PMC4575699; doi:10.1007/s00429-014-0881-y)
Supplement: Supplementary file 1 — Supplementary material 1 (PDF 126 kb) [file 429_2014_881_MOESM1_ESM.pdf]

	CSTinf	CSTmid	CSTsup	STT	OR	ALIC
σ_+ (%)	1.3 ± 0.2	1.6 ± 0.3	1.6 ± 0.2	0.9 ± 0.3	1.0 ± 0.1	1.1 ± 0.1
σ_- (%)	3.9 ± 0.5	3.1 ± 0.1	3.2 ± 0.2	5.7 ± 0.9	5.0 ± 0.5	5.0 ± 0.2
	EC	AF	SLF	ILF	UF	FOF
σ_+ (%)	1.0 ± 0.1	1.1 ± 0.1	0.7 ± 0.1	0.9 ± 0.1	0.7 ± 0.1	0.8 ± 0.1
σ_- (%)	0.8 ± 0.4	4.8 ± 0.4	4.9 ± 0.5	6.1 ± 0.4	9.0 ± 0.1	6.8 ± 0.5
	FX	CGinf	CGsup	CCg	CCb	CCs
σ_+ (%)	0.6 ± 0.1	0.9 ± 0.1	0.9 ± 0.1	0.6 ± 0.2	0.8 ± 0.2	0.9 ± 0.2
σ_- (%)	8.4 ± 0.7	6.2 ± 0.4	6.1 ± 0.4	8.9 ± 0.3	6.5 ± 0.1	5.9 ± 0.5

Online Resource 1

Calculation errors of the Mahalanobis distance for the different bundles. These errors suggest that Mahalanobis distance is usually slightly underestimated, on average by 6.0±1.8%. See Fig.2 for abbreviations.
