# Supplementary material for: Multi-parametric evaluation of the white matter maturation
Source: Brain Struct Funct. 2014 Sep 3;220(6):3657–72. doi: 10.1007/s00429-014-0881-y (PMC4575699; doi:10.1007/s00429-014-0881-y)
Supplement: Supplementary file 2 — Supplementary material 2 (PDF 167 kb) [file 429_2014_881_MOESM2_ESM.pdf]

	CSTinf	CSTmid	CSTsup	STT	OR	ALIC
M	**22**	**28**	13	**27**	**26**	15
FA	26	--	36	--	--	--
<D>	98	51	15	--	40	43
$\lambda_{\|}$	--	164	40	--	45	66
$\lambda_{\perp}$	--	52	14	--	48	71
qT1	100	38	27	--	32	**11**
qT2	--	36	**10**	--	39	16
	EC	AF	SLF	ILF	UF	FOF
M	**5**	**14**	24	**20**	**24**	14
FA	33	40	61	--	--	--
<D>	33	32	21	32	64	31
$\lambda_{\|}$	58	62	**10**	34	50	28
$\lambda_{\perp}$	24	31	30	31	75	32
qT1	100	38	27	--	32	**11**
qT2	--	36	**10**	--	39	16
	FX	CGinf	CGsup	CCg	CCb	CCs
M	**27**	**5**	**9**	**24**	**4**	**8**
FA	--	--	--	79	--	--
<D>	--	28	38	--	94	52
$\lambda_{\|}$	--	--	33	--	--	71
$\lambda_{\perp}$	--	22	49	--	84	48
qT1	32	14	15	27	21	12
qT2	--	11	12	--	--	35

Online Resource 2

Prediction errors (in %) of the maturational age in the leave-one-out validation for different bundles and different parameters. Unlike univariate approaches, Mahalanobis distance could be used to make predictions for all bundles, and provided smaller errors for 14 out of 18 bundles (except for CSTsup, ALIC, SLF, ILF) in comparison with other parameters (“--” indicates that the linear regression could not be performed). Bold numbers highlight the smallest prediction errors for each of the bundles. See Fig.2 for abbreviations.
